# The Combined Effect of IgG and Fe Supply and Feeding Management on Growth Rates of Calves on Eight Commercial Dairy Farms in Germany

**DOI:** 10.3390/ani12070850

**Published:** 2022-03-28

**Authors:** Odile C. Hecker, Iris Schröter, Andreas Rienhoff, Anne Thönnissen, Elena Meininghaus, Sabrina Burkert, Marcus Mergenthaler, Marc Boelhauve

**Affiliations:** Department of Agriculture, Westphalia University of Applied Sciences, 59494 Soest, Germany; hecker.odile@fh-swf.de (O.C.H.); schroeter.iris@fh-swf.de (I.S.); rienhoff.andreas@fh-swf.de (A.R.); thoennissen.anne@fh-swf.de (A.T.); meininghaus.elena@fh-swf.de (E.M.); burkert.sabrinamaria@fh-swf.de (S.B.); mergenthaler.marcus@fh-swf.de (M.M.)

**Keywords:** immunoglobulin G (IgG), iron, calf, colostrum, management, weight gain

## Abstract

**Simple Summary:**

Well-thought-out calf rearing is essential to produce long-lived and productive cows for future dairy herds. The first days of a calf’s life are a very important period. It is known that calves that are adequately supplied with colostrum during this period have sufficient antibodies. They gain weight faster and fall ill less frequently. Identifying other management factors that have positive effects on calf growth is the subject of this study. To investigate this, 123 Holstein calves on eight conventional dairy farms were followed from birth to 50 days of life in spring 2017, and the influence on the animals’ weight gain due to management factors such as timing and supply of colostrum, iron supplementation, feeding regime and microbial load at first feeding was analyzed. The results show that, in addition to an adequate level of antibodies in the colostrum fed to the calves, the iron concentration in the calves’ blood and the feeding regimen play an important role in the calves’ weight gains and thus in calf rearing.

**Abstract:**

During the first days of a calf’s life, the foundations are laid for successful growth and thus also for the later performance of the cows. The aim of the present study was to analyze the impact on the weight gain of newborn calves due to important management factors related to colostrum supply, iron supply, feeding regime and microbial load at first feeding. In spring 2017, information of 123 Holstein calves were analyzed with regard to the colostrum supply and management factors on eight commercial dairy farms located in Germany. Additionally, blood samples of newborn calves were analyzed for total immunoglobulin G (IgG) and serum iron content. Furthermore, Brix analysis and analysis of contamination by *E. coli* were performed on first colostrum samples from teats or buckets. Average daily weight gain of calves at days 14 and 50 was calculated. The colostrum IgG was estimated by Brix refractometer. The volume of initial colostrum supply and the time between birth and colostrum intake significantly (*p* < 0.05) influenced the serum IgG concentration. The serum IgG concentration, the serum iron concentration and the feeding regimen (restrictive or not restrictive feeding) had a significant effect (*p* < 0.05) on daily weight gains. In conclusion, this study shows that, in addition to a sufficient supply of immunoglobulins, other aspects, such as an adequate colostrum, iron and milk supply, play an essential role in calf growth rates.

## 1. Introduction

Essential parameters for a progressive dairy farm are the rearing of long-lived and productive dairy cows. In their recent review, Hammon et al. point to intolerably high mortality and morbidity rates during calf rearing, which contradicts the goal of rearing long-lived and productive dairy cows [1]. A study on German cattle mortality reveals that cattle at <6 months of age account for 50% of all deaths (excluding slaughter for human consumption) [2]. These values illustrate the importance of this very sensitive and critical period from birth to weaning of calves. Since calves are usually immediately separated from their dams, appropriate calf management by farmers during this early phase is essential to positively influence the later performance of the animals. Long-term benefits of optimal care during the rearing period on the future life of dairy cows include improved rate of weight gain, reduced mortality in the post-weaning period, reduced age of puberty onset and improved lifetime productivity [3,4,5]. Particularly, feeding management during the neonatal and preweaning period affects the success of calf rearing, health and performance in later life [6,7,8]. Therefore, attention should be given to colostrum management and appropriate milk feeding policies of dairy calves to meet their needs for optimal growth and development.

Calves are born agammaglobulinemic, and for establishing passive immunity they need to ingest and absorb adequate amounts of colostral immunoglobulins [9]. Numerous studies analyzed the importance of passive transfer of colostral immunity in calves on morbidity, mortality rate and production [10,11,12]. Inadequate colostral IgG absorption in calves is termed “failure of transfer of passive immunity” (FTPI). To avoid FTPI, a successful colostrum management program includes the quantity of colostrum provided, the time of the first feeding and the quality of the colostrum fed as determined by IgG concentration [13,14,15]. The time of the first milking has an influence on the level of IgG content in the colostrum [16]. The IgG concentration in the milk can be measured with a Brix refractometer [17].

Long-term effects of the rearing period are not exclusively correlated with colostral immunoglobulin passive transfer in calves. For optimal growth and development, other factors such as appropriate provision of iron are also important. The iron requirement of animals varies according to age, sex and condition of the organism [18]. It is generally accepted that the iron requirement of young animals is greater than those of adult ruminants and is estimated to be about 100 mg per calf per day [19,20]. Transient iron deficiency in newborn calves can lead to clinical and subclinical symptoms of anemia and increased susceptibility to infections [21]. Despite the importance of iron for the normal growth of calves and their resistance to infections, cow’s milk is low in iron (about 10 ppm), and it may not fully supply the requirements of newborn calves [22]. Although calf milk replacer (CMR) contains greater levels of iron than cow’s milk and thus may meet the needs of rearing calves, it should be noted that numerous studies indicate that the administration of iron to calves (orally or parenterally) provides an increase in hematological parameters and better growth [20,22,23,24].

Furthermore, the collection, handling, and storage of colostrum carries the risk of microbial contamination [25]. Especially as collection is often by milking techniques that are different from routine hygienic practices for milking, instrumentation and milk storage. Moreover, storage until the colostrum is fed to the calf may be at room temperature [25]. Therefore, a large number of calves are at high risk of receiving colostrum of poor quality with high bacterial loads [26]. Microbial contamination of colostrum has the potential to cause diseases and could reduce the efficiency of IgG absorption [27]. Thus, cleaning of milking and calf-feeding equipment and udders before the start of milking and before storing and feeding colostrum are necessary to improve colostrum quality and to reduce the risk for morbidity and mortality in preweaning calves [28].

The objectives of this study were to bring together the major aspects of primary care and appropriate milk feeding by analyzing the effects of IgG and iron supply, feeding regime (amount and duration of whole-milk feeding) and microbial load of colostrum at first feeding on daily weight gain of preweaning calves under practical conditions in commercial German dairy farms. In detail, the study aims to (i) analyze to what extent the Brix value of the colostrum, the amount of the first colostrum administration and the time interval between birth and the first colostrum feeding affect the serum IgG concentration; (ii) analyze the effect of oral iron supplementation with the first colostrum intake on serum iron (Fe) concentration; (iii) examine the predictors of growth (serum IgG, iron status and management factors) within the first 14 days. Furthermore, the correlation between daily weight gain in the first 14 days and further weight development (day 50) was investigated.

## 2. Materials and Methods

In the period from March to August 2017, the management of newborn calves was recorded in eight dairy farms in north Rhine–Westphalia (Germany). These were exclusively family farms with the residential house in the immediate vicinity. Herd size ranged from 90 to 210 adult dairy cows. Newborn calves remained on the farm for at least 14 days, and the female offspring for longer as needed. At the beginning of the project period, each farmer filled out a questionnaire that asked about the size of the farm, as well as the general housing conditions of the calves. In addition, there was an information meeting where the farmers were informed about the process of the project and gave their consent to the project. At this meeting, the farmers also received standardized templates for documenting the individual calf management aspects analyzed in this study.

Detailed information of individual calves with regard to the birth process, including dam identification number, calf identification number, birth date and time, time and amount of colostrum feeding, the milk feeding regime (restrictive, ad libitum; duration of whole-milk feeding) and sex and number of calves born (single/twin) were recorded by the farmers on predefined templates from day 1 (birth) up to day 14 ± 1.5. In addition, each calf underwent a clinical examination by the latest on the third day of life (median 1.5 days of life) by a veterinarian and was routinely tested for serum IgG and Fe concentrations. Clinical examination routinely included assessment of heartrate, respiratory rate, temperature and lungs (as an important organ system), in addition to an assessment of the calves’ general condition.

On all farms, calves were born in calving pens and were usually separated from their mothers immediately after birth. However, the specific time period from birth to separation differed among calves depending on the time of birth (e.g., longer time periods for births that occurred at night). Farmers recorded the time-points of separation from the dam and if the calf had suckled. Calves that had been suckling at the dam were excluded from the analysis of variables associated with serum IgG concentration. During the following individual housing, calves were housed in individual calf igloos in five farms, while three farms used individual calf pens.

On each farm, colostrum was harvested with the milking equipment available on the respective farm. Depending on the time of birth, colostrum from the dams was milked after birth by the farmers as soon as possible. The volume and time-points of first milking and feeding were recorded by the farmers.

At subsequent milk feedings, the calves received warm milk (35 °C to 40 °C) by teat bucket twice daily. Milk provision was either restrictive or ad libitum. During restrictive offer, calves received three liters of milk twice a day, after which the bucket was removed. In contrast, in ad libitum offer, the bucket was filled with milk twice a day to the maximum of 6 L and remained attached to the igloo or calf pen. Depending on the feeding practice, the calves were fed twice daily with a commercial milk replacer (calf starter) either after five or 14 days of whole-milk feeding. The energy content of the calf starter is 18.5 MJ/kg dry matter, and it consists of 22% crude protein,18% crude fat, 0.1% crude fiber, 6.4% crude ash, 0.81% Ca, 0.58% P and 0.43% Na as analytical components based on the manufacturer’s instructions (Denkamilk Royal 30.I, Denkavit, Warendorf, Deutschland). The calf starter also contained the following additives: 80 mg/kg Fe, 9 mg/kg Cu, 29 mg/kg Mn, 100 mg/kg Zn, 0.5 mg/kg I, 0.2 mg/kg Se, 25,000 IE vitamin A, 4010 IE vitamin D3 and 150 IE vitamin E. Participating farmers offered their calves either six liter (restrictive) or ad libitum (bucket filled twice a day to the maximum) CMR per day with a mixing rate of 125 g of milk solids per liter. The milk replacer formula contained 80 mg/kg of iron in the form of iron sulfate. According to normal farm routines, all aspects of the order on the protection of animals and the keeping of livestock (German designation: TierSchNutztV) with regard to feeding, including access to water and group housing after 14 days, were considered.

Postnatal, 17 calves of one farm received iron in the form of an oral supplementary feed containing 2505 mg/kg Fe (Eisen-(II)-sulfat Monohydrat) (Denkacare Vitaladd, Denkavit, Warendorf, Deutschland) twice daily. The oral complementary feed was included with the colostrum feeding in a dose of 5 g per liter. Subsequently, the complementary feed was added to whole-milk feeding in a dose of 10 g per liter for a period of one week.

Colostrum samples were collected by the dairy person from individual cows at the first milking after calving from teats or buckets with a 100 mL sterile cup immediately prior to the first feeding of the calf. Each sample was dated and marked with the specific identification number of the cow and calf. Samples were stored at −20 °C until transport and analyzation. Samples were thawed at room temperature, and analyses of percent Brix were performed using a standard digital Brix refractometer (DR101-60 Digitales Handrefraktometer, A. KRÜSS Optronic GmbH, Hamburg, Germany).

In addition, a decimal dilution for microbiological examination of each sample was prepared for analysis of *E. coli* onto Rapid Enterobacteriaceae Escherichia Coli Coliform Agar (REBECCA; Biomérieux, Nürtingen, Germany). The plates were incubated at 37 °C in an atmosphere of ambient air. After 24 h of incubation, bacterial colonies were counted. For analyses, plate counts were categorized as no growth (*E. coli* absent) or growth (*E. coli* present).

The birthweight of the calves was determined within 12 h after birth using a personal scale (Beuer GS212, Beuer, Ulm, Germany). All calves were weighed at 14 and 50 days using a floor scale (Kern EOS, Kern & Sohn GmbH, Balingen, Germany) (14 ± 1.42 day; 50 ± 3.19 day). Average daily weight gain was computed by subtracting birthweight from the bodyweight obtained at the time of measurement and dividing this difference by day of age.

Total IgG concentration and serum iron content were obtained from a single blood sample of each calf, which was taken from the jugular vein by farm-supervising veterinarians by the third day of life at the latest (median 1.5 days of life) and were sent to a veterinary laboratory (Laboklin, Bad Kissingen, Germany) for examination. Samples were tested according to common laboratory methods. Briefly, total IgG concentration was determined by capillary electrophoresis via separation of serum proteins using the Minicap device (Sebia, Fulda, Germany), and serum iron content was measured by the Ferrozine method. Fe^3+^ ions are released from the FE-transferrin complex and reduced to Fe^2+^, which reacts to form a colored complex. Color was measured using the Cobas 8000 c701 instrument (Roche Deutschland Holding GmbH, Grenzach–Wyhlen, Germany).

Overall, 141 calf serum samples were available from Holstein calves on eight dairy farms. Calves that died during the study period for various reasons were included in the respective analyses if the data situation allowed. Data of eighteen samples were excluded from the analyses due to hemolysis of the blood sample. Of these 123 remaining calves included in the study, 72 were female and the remaining 51 calves were male. Six of the calves were twins. Furthermore, 32 calves due to having been suckling at the dam and 10 due to inconsistencies in the farmer’s records were excluded from the analysis of variables associated with serum IgG concentration. In the analysis of average daily weight gain (ADG) of calves within the first 14 days compared with ADG within 50 days, calves suckling at the dam were included, but 34 calves aged more than two weeks were sold by the farmers as usual practice. Hence, the analysis was performed with the complete number of samples available (*n* = 79). Individual calf data were stored and examined for plausibility with Microsoft Excel™. Data were analyzed with SPSS (IBM Corp. Released 2017. IBM SPSS Statistics for Windows, Version 25.0. Armonk, NY, USA).

An analysis of variance was performed to analyze whether the serum IgG content in the blood depends on feeding parameters. The Brix value was used as an indicator of the IgG content in the milk. Independent variables included Brix (in two classes), time from birth to colostrum intake (in four classes) and volume of initial colostrum uptake (in three classes). For post hoc tests, Bonferroni correction was applied. Uptake of initial colostrum was categorized as (1) uptake of >3 L; (2) uptake of >2 to ≤3 L; or (3) uptake of ≤2 L, and time from birth to colostrum intake was categorized as (1) ≤1 h; (2) >1 to ≤2.5 h; (3) > 2.5 to ≤ 4 h; or (4) >4 h. Results of the Brix refractometric measurement were categorized according to the threshold value (Laboklin, Bad Kissingen, Germany) as (1) Brix score <22%, insufficient IgG concentration in the colostrum sample and (2) Brix score ≥22%, sufficient IgG concentration in the colostrum sample. The effect of iron supplementation on serum iron levels was assessed by Welch test. The influence of sex, blood and management parameters on 14-day daily weight gain of calves was tested using analysis of variance. The parameters were gender (male, female; twins were excluded from the analysis), serum Fe concentration categorized as (1) <20 mmol/L or (2) ≥20 mmol/L, serum IgG concentration categorized as (1) <800 mg/dL or (2) ≥800 mg/dL, milk feeding (restrictive, ad libitum), whole-milk feeding (5 days or 14 days) and *E. coli* in the first colostrum meal of the calf (present or absent). The categorization of Fe and IgG was performed according to the thresholds recommended by the laboratory for the corresponding test method. A simple Pearson correlation was calculated to show the relationship between the ADG within the first 14 days and the ADG within 50 days of age of the calves.

## 3. Results

### 3.1. Predictors of Serum IgG Concentration

The mean IgG concentration of the calf serum samples was 1425 ± 689 mg/dL (minimum, 300 mg/dL; maximum, 3130 mg/dL). The serum IgG concentration was <800 mg/dL in 34 of 81 calves, thus 42.0% of calves had a serum IgG concentration below the recommended limit (Laboklin, Bad Kissingen, Germany).

The influence of the independent variables on the dependent variable (serum IgG) is shown in Table 1. The analysis comprises data from 81 calves, the predictors included are Brix, volume and time of first colostrum feeding after birth.

Farmers indicated that the cows were milked for the first time within 2.68 ± 2.63 h after birth. The variation in time to colostrum collection, reported by the farmers, ranged from 0 to 8.5 h, with 55/81 (67.9%) of farmers collecting the colostrum within the first 2 h after birth. The mean percent Brix of colostrum samples was 22.1% (SD ± 5.2) with a range of 9.0 to 33.1%. Values were ≥22% Brix for 43/81 (53.1%) samples. A considerable number of the calves (91%) were fed the first colostrum within 4 h after birth, with 74% of calves being fed even within 2.5 h and 35.8% even within 1 h after birth. In this study, 47% of calves received 2 to 3 liters and 21% of calves received more than 3 liters of the first colostrum, whereas 32% of calves consumed less than or equal to 2 liters of first colostrum.

The quality of colostrum fed (Brix), the volume of colostrum fed and the timing of the first feeding after birth were significantly associated with the calf serum IgG concentration (Table 1). In the analysis of variance, colostrum with a value of ≥22% Brix was associated with greater serum IgG levels than colostrum of less quality (<22% Brix). In addition, the timing of the initial colostrum intake and the volume of colostrum fed were significantly associated with serum IgG concentrations. The estimated marginal means of serum IgG concentration were highest in calves that received initial colostrum within the first hour after birth and decreased with increasing time. Uptake of more than 3 liters of initial colostrum was also associated with a high estimated marginal mean serum IgG concentration, whereas lesser volumes of colostrum uptake resulted in decreased estimated marginal means of serum IgG concentration.

### 3.2. Serum Fe Concentration

**Figure 1:**
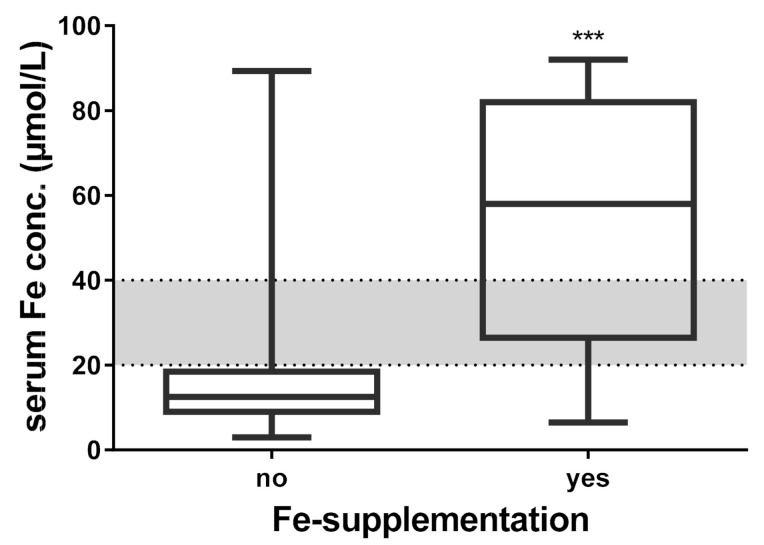
Serum Fe concentration of calves with and without oral iron supplementation (*n* = 123, calves receiving supplementation: no, *n* = 106; yes, *n* = 17), normal range highlighted in grey, *** *p* < 0.001.

In the current study, serum analysis showed serum Fe concentrations equal to or less than 20 µmol/L (threshold according to Laboklin, Bad Kissingen, Germany) in 69.9% of calves. Iron supplementation was given to 17 (13.8%) of 123 calves. The mean serum Fe concentration was 15.44 ± 11.30 µmol/L for calves without iron supplementation and 54.50 ± 27.48 µmol/L for calves with supplementation. There is a statistically significant impact of iron supplementation on the serum iron concentration of the calves (Welch-test; *t* (16.88) = −5.756, *p* < 0.001) in Figure 1. There was no significant influence of the volume of first colostrum ingested on the serum Fe concentration of the calves (Welch-test; F (2; 52.08) = 1.841, *p* = 0.169).

### 3.3. Predictors of Growth

Farmers provided CMR for 52.2% of the calves after five days of whole-milk feeding, whereas the remaining calves received CMR after 14 days. The drinking supply was restrictive for 61.9% and ad libitum for 38.1% of the calves. *E. coli* were present in 17.7% of initial colostrum samples, whereas it was absent in 82.3% of samples. Statistically significant differences (*p* ≤ 0.05) in ADGs were found between the group of calves with serum Fe concentrations greater than or equal to 20 µmol/L and between the group of calves with serum IgG concentrations greater than or equal to 800 mg/dL than below these thresholds (Table 2). In addition, the group of calves with ad libitum milk feeding had statistically significantly greater ADGs in comparison to calves fed restrictively; further, the group of calves fed whole milk for 14 days had greater ADGs than the group of calves fed whole milk for 5 days. The difference between positive and negative *E. coli* in the initial feed of colostrum was not statistically significant with regard to the ADGs. Likewise, no differences in the ADGs were found between male and female calves. The highest ADGs were observed after whole-milk feeding for 14 days (estimated marginal mean of 0.88 kg ADG) and serum IgG concentrations greater than or equal to 800 mg/dL (estimated marginal mean of 0.83 kg ADG) (Table 2), followed by the serum Fe concentration (0.81 kg ADG) and ad libitum milk feeding (0.81 kg ADG).

### 3.4. Calf Development

Of 113 calves whose development was followed by weight data, one died within the first 14 days. The calf was euthanized the day after birth due to respiratory problems. Subsequently, three more calves died in the first 50 days of life due to Clostridia infection, malnutrition and unknown cause. That is, the mortality rate of calves was 0.88% in the first 14 days and 3.54% in the first 50 days.

**Figure 2:**
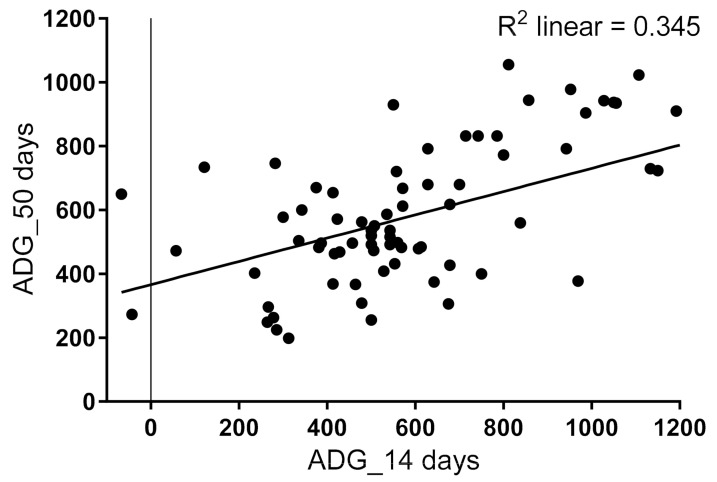
Average daily weight gain (ADG) of calves within the first 14 days compared with ADG within 50 days (*r* = 0.588; *p* < 0.001; *n* = 79).

At birth, calf weights were 41.1 ± 5.6 kg (*n* = 113), increasing to 51.3 ± 7.7 kg within 2 weeks, Figure 2. The mean weight was 70.6 ± 11.9 kg at 50 ± 3 days (*n* = 79). The ADG between 1 and 14 days of age was 0.71 ± 0.43 kg. Overall, 29 of 113 of calves (25.7%) grew <0.5 kg/day between 1 and 14 days. Two calves even lost weight during this period of time. In contrast, 49 of 113 calves (43.4%) gained weight more quickly, with growth rates at or above 0.7 kg/day. Greater daily weight gain within the first 14 days was positively related to further weight development of the calves at day 50 (*p* < 0.0001; *n* = 79; Figure 2).

## 4. Discussion

Inadequate colostral IgG adsorption in calves often leads to decreased performance and longevity and increased risk of diseases and mortality [29]. In the present study, the mortality rate was 0.88% in the first 14 days and 3.54% in the first 50 days, which is comparatively low since other studies have reported mortality rates of 8 to 25% [29]. However, reducing mortality rates alone cannot be the only goal of farmers in calf care. Rather, good care of calves in the first days of life aims to positively influence subsequent lifetime performance of the animals, as optimal care of the animals has been shown to positively alter organ growth in terms of metabolic programming [30]. Our results show that the calves which were adequately supplied with IgG and Fe and which received ad libitum feeding management with whole-milk feeding for 14 days outperformed other, less well-fed calves in terms of daily weight gains. The results may be used by practicing farmers as an incentive to adjust their on-farm colostrum management, which might increase the lifetime performance of their animals.

In this context coherent colostrum management in all areas is necessary to increase development in terms of daily weight gain of young calves while preventing health disorders [10,29]. Most previous studies on calf development have focused either on failure of passive transfer, on nutrition or on iron supply [9,20,31]. The present study combined important management factors and measured their resulting impact on calf growth rates on commercial farms applying their usual management practices in Germany.

Although this was a convenient sample of eight Holstein herds, the study should be reasonably generalized to other commercial dairy herds with similar structures and management practices. However, farms were chosen based on their willingness to participate, and, therefore, self-selection bias cannot be excluded. Overall, the data obtained regarding ADG point to good practices in calf management. Intensive interaction with the farmers was an important issue during the project course. Therefore, we assume that calf were provided above-average care. A major concern of dairy farmers is to achieve a sufficiently high concentration of IgG in serum within the first day of life to ensure the health of newborn calves [10]. In the present study, the concentration of IgG measured in the serum of calves following administration of colostrum was 1425 mg/dL. This level is comparable to mean calf-serum IgG concentrations reported in previous studies [11,13]. Based on the results of the serum IgG concentration, 42% of calves had failure of passive transfer of immunity (FTPI). The rate was similar to another German study of 1656 serum samples of calves where 40% of the calves had FTPI [32]. Colostrum of poor quality was the main risk factor associated with FTPI in a previous study [16]. The results of the present study indicate that approximately half of the colostrum samples fed to newborn calves had Brix less than 22% and therefore were suboptimal in quality by international standards [14,33]. This is widely comparable to results of a study from Australia, where 46.7% of colostrum samples were reported to have Brix <22% [26]. In contrast, studies from the United States of America reported only between 16 and 34% of colostrum samples with Brix <23% [17,34]. Factors such as the calving-to-colostrum-collection interval, volume at first milking, season, herd-specific factors and parity of the dam influence the IgG content of colostrum [16,35]. It was shown that the calving-to-collection interval is a critical point, with colostrum collected within 2 h after parturition containing the highest IgG content [36]. In the current study, the variation in time to colostrum collection showed a wide range from 0 to 8.5 h, with more than 30% of farmers collecting the colostrum after the recommended period of two hours after birth. As it was also suggested that colostral IgG concentration is attributable to endocrine regulation or genetic variation of the appropriate mammary transporters it is possible that genetic variation of the dams in the study sample contributed to the differences in IgG [37]. Consequently, 49.1% of the calves in our study were in the unfavorable situation of receiving colostrum with poor IgG content. Other factors in the management of the calves could have compensated or strengthened this adverse effect. Besides the colostral IgG concentration, serum IgG concentration is influenced by the time interval from birth to colostrum intake as well as by the volume of initial colostrum intake [9]. Immunoglobulin transfer across the gut epithelium is optimal in the first four hours postpartum and begins to decline rapidly after 12 h postpartum [9].

In the present study, 42 of 113 calves (37%) were born between 9:00 pm and 7:00 am. This time could be critical because farmers typically monitor calving females less closely at these times of day. This may increase the time from birth to first colostrum administration. However, in the study population, 91% of calves received colostrum in the first 4 h after birth. Hence, it can be concluded that farmers are aware of the importance of an early supply of colostrum. In addition, the volume of the first colostrum feeding influences the serum IgG concentration [10]. It is recommended that Holstein calves be fed 3–4 liters of colostrum at first feeding [10], and, in the present study, 68% of calves consumed at the first administration more than 2 liters and 21% of calves more than 3 liters of colostrum. However, 28% of calves suckled the dam, which is the least preferred approach, because on the one hand it can lead to delayed suckling and failure to control quantity and quality of ingested colostrum with the consequence of greater rates of failure of passive transfer [38], and on the other hand, it may increase the calf’s risk for exposure to pathogens from the dam or her environment. Therefore, it is currently recommended that the calf be removed from the dam within 1 to 2 h of birth and then hand-fed a known volume of colostrum [39]. However, even when hand-feeding with a teat bucket or bottle, perfect colostrum quality can only be achieved if the colostrum is free of bacteria. Bacterial contamination of colostrum may occur at the milking process, during storage or during feeding [40]. It was reported that high levels of bacteria in colostrum, and particularly coliform bacteria, may bind free Ig in the gut lumen and/or directly block uptake and transport of Ig molecules across intestinal epithel cells, thus interfering with passive transfer [41]. Moreover, the exposure of pathogenic bacteria in colostrum is a concern because it could cause diseases such as diarrhea or septicemia [42]. In the present study, 18% of the colostrum samples from the drinking buckets were contaminated with *E. coli*. This should be avoided at all costs, as contamination of colostrum with microbial pathogens may result in acute or chronic disease [28]. In addition, other studies showed an adverse effect of bacteria in colostrum on serum IgG levels and the efficiency of IgG absorption [27,43]. The fact that we did not observe a significant effect of *E. coli* on the estimated marginal means of ADG in our study may be due to the fact that only the presence and not the absolute amount of *E. coli* was considered. It should therefore not be assumed from our study that good drinking hygiene is not needed during primary care. In total, emphasis should be taken on the importance of minimizing colostrum contamination by properly prepping udders before harvesting colostrum, milking into a clean, sanitized bucket and handling colostrum using clean, sanitized storage or feeding equipment.

All of the above-mentioned factors influenced the serum IgG concentration of the study calves, resulting in an adequate supply with serum IgG concentrations greater than or equal to 800 mg/dL in 69.9% of the calves. Greater daily gains were achieved by calves with adequate serum IgG concentrations. However, serum IgG concentration is not the only factor that should be considered for greater daily gains and optimal calf rearing. Besides serum IgG concentration, other variables are important for optimal calf growth. In our study, growth rates at 14 days were below optimal for many preweaned calves, with 26% growing at <0.5 kg/day. As an ADG of 1 kg/day is achievable on an ad libitum milk supply [8], ADG of 0.5 kg/day was considered as a level of growth restriction that would be expected to reduce calf welfare [44,45]. On the other hand, 43% of calves grew at >0.7 kg/day at 14 days, which is a greater proportion of calves than reported by a study in southeast England where only 30% grew at >0.7 kg/day [45] and 25% even grew at >1.0 kg/day. It was shown that for heifer calving at <26 months (which is associated with increased productivity and longevity), ADG rates between 1–6 months of more than 0.7 kg/day were needed [46]. Due to different age structures of the different studies, the numbers are difficult to compare.

Restriction of the milk ration for young calves is common in calf-rearing systems. Therefore, it is not astonishing that 62% of calves in the present study received restricted milk feeding, which resulted in significantly decreased estimated marginal means for daily gain (Table 2). Additionally, about half of the animals received whole milk for only a five-day period and were then switched to a commercial milk replacer. In these animals, the estimated marginal means of weight gain were significantly decreased compared to animals receiving whole milk for a duration of two weeks. It may be concluded that the metabolisable energy (ME) of the milk replacer was too low to achieve greater daily gains. Considering that calves on ad libitum feeding consume approximately 8 liters/day from birth to weaning at 60 days of age [47], this corresponds to an equivalent of the metabolisable energy from about 1.1 kg/day of a typical milk replacer (22% protein, 18% fat and 7% ash). The restrictively fed calves, however, received (in accordance with the manufacturer’s feeding instructions) only 0.75 kg/day of the commercial milk replacer. In order to achieve greater daily gains and thus positive effects on the subsequent performance of the future dairy cow, a sufficient energy supply must be ensured either by adjusting the ration of the milk replacer accordingly or via whole-milk feeding. It also became apparent that greater daily weight gains within the first 14 days were positively related to further weight development of the calves at day 50.

Since an iron deficiency is associated with numerous clinical signs, including anemia, reduced growth and increased occurrence of diseases, numerous studies recommend an additional iron supply to newborn calves to further bolster the positive effects of an adequate serum IgG concentration and an optimal milk feeding management [19,20,23,24]. The iron supplementation can be given via the mouth and simply mixed with colostrum or administered as an iron injection. Sufficient iron content in the diet is necessary for the production of red blood cells (RBC) and hemoglobin (Hb) and thereby for anemia prevention [20]. Basically, the initial serum iron content was below the threshold in 69.9% of the study calves. Only one calf that received iron supplementation had a serum iron content below the threshold. The concentration of Fe in the blood of young calves can vary by a wide range, from less than 10 mol/L to as high as 41 mol/L [48,49]. Therefore, not all newborn calves have iron levels below the threshold, per se. Rather, over the first few days of life a progressive reduction in serum Fe concentration occurs, thus many authors point to the need for supplementation [18,20,50]. Calves receiving iron supplementation had significantly greater blood iron levels (*p* < 0.001). The iron content of whole milk is 0.5 mg/L, which is very low [19]. Since a supply of 100–150 mg of iron per animal per day is recommended for the preruminant calf, this amount cannot be covered by whole milk alone [19,51]. Therefore, it is recommended to supplement with whole-milk enhancer (vitamins and trace elements). In the present study, calves received a commercial calf milk replacer containing 80 mg/kg iron after either five or 14 days. With an intake of calf milk replacer of 10 L per day, the calves were thus sufficiently supplied with iron. Furthermore, toxic effects of high iron intake in ruminants have also been described in the literature: e.g., excessive iron intake can lead to the production of oxygen radicals and expose sensitive tissues to oxidative stress [49]. Adult cows, with 24 mg/kg of dry matter, require much less iron than calves [51]. In addition, some ruminant feedstuffs, such as alfalfa and corn gluten feed, contain high levels of iron [52]. Since newborn calves initially only drink milk, the absorption of iron through feed is not available to them. If the commercial iron additives are used properly, toxic effects are not expected. Additionally, total weight gains and mean daily weight gains were shown to be greater in iron-supplemented calves [20]. In the present study, the estimated marginal means of weight gain during the first two weeks of life for calves that had iron levels ≥20 mmol/L were significantly greater than for calves that were undersupplied with iron. Since only one farmer supplemented iron in all his calves, the effect of iron on estimated marginal means of weight gain cannot be separated from a farm effect. Based on results from other studies, each of which showed better weight gain through iron supplementation, it can be assumed in the present study that the greater estimated marginal means of weight gain were due to iron supplementation [18,20,50]. In addition, the farms participating in the study were comparable in terms of management and housing conditions.

The development of the rearing calves in the early rearing period is of great importance for the further development of the animals into top-performing dairy cows. Because calves are born agammagluteinemic, and immunglobulin production has yet to develop, FTPI is a major focus of farmers in the care of dairy calves, and colostrum management is described as the single most important management factor in determining calf health and survival [42]. The results presented here show that a combination of management factors have a positive effect on calf development and that this also includes adequate Fe supply and continued feeding management.

## 5. Conclusions

In conclusion, this study shows that, in addition to a sufficient supply of immunoglobulins, other aspects such as an adequate milk and iron supply play an essential role in calf rearing. The results allow farmers to optimize their calf management, contributing to an increase in the short- and long-term health and performance of the animals on their farm.

## Figures and Tables

**Table 1:** Variables associated with serum IgG concentration at 1–4 day of Holstein calves on eight commercial dairy farms (*n* = 81).

Variables	Factor Level	Estimated Marginal Means (Serum IgG Concentration mg/dL)	SE	*n*	F	*p*	Partial η2
Brix (%)	˂22	1097.3	106.5	38	15.709	<0.001	0.175
	≥22	1615.8	98.6	43			
Volume of Initial Colostrum (L)	≤2	1208.9	112.6	26	3.486	0.036	0.086
	>2 to ≤3	1225.7	108.9	38			
	>3	1635.1	153.6	17			
Time from Birth to Initial Colostrum Intake (h)	≤1	1701.9	106.9	29	3.932	0.012	0.137
	>1 to ≤2.5	1407.5	108.8	31			
	>2.5 to ≤4	1383.8	155.6	14			
	>4	933.0	222.1	7			

IgG: Immunoglobulin G; SE: Standard Error; F: F-ratio.

**Table 2:** Variables associated with calf average daily weight gain (ADG) (kg/day) between 1 and 14 days of age of 113 Holstein calves on eight commercial dairy farms.

Variables	Factor Level	Estimated Marginal Means (ADG kg)	SE	*n*	F	*p*	Partial η2
Sex of the Calf	male	0.72	59.24	47	0.096	0.757	0.001
	female	0.70	49.84	66			
Serum Fe Concentration	<20 mmol/L	0.60	48.82	78	9.978	0.020	0.086
	≥20 mmol/L	0.81	63.03	35			
Serum IgG Concentration	<800 mg/dL	0.58	64.37	34	14.00	<0.001	0.117
	≥800 mg/dL	0.83	45.35	79			
Milk Feeding	Restrictive	0.60	52.85	70	9.363	0.003	0.081
	ad libitum	0.81	59.86	43			
Whole-Milk Feeding	5 days	0.54	57.81	59	28.39	<0.001	0.211
	14 days	0.88	52.66	54			
*E. coli*	present	0.66	77.33	20	0.978	0.325	0.009
	absent	0.75	35.38	93			

SE: Standard Error; F: F-ratio.

## Data Availability

The data presented in this study is available on reasonable request from the corresponding author.
